# Refractory bile reflux following one-anastomosis gastric bypass: A case report and literature review on surgical management

**DOI:** 10.1016/j.ijscr.2025.111642

**Published:** 2025-07-09

**Authors:** Andres Fontaine-Nicola, Paula Cambuli-Bianchi, Kaiser O'Sahil Sadiq, Gabriel Carrizo, Pablo Omelanczuk

**Affiliations:** a Department of Surgery, Division of Minimally Invasive Surgery, University of California San Diego, San Diego, CA, USA; b Department of Surgery, Division of Esophageal, Gastroduodenal and Bariatric Surgery, Italian Hospital of Mendoza, Mendoza M5519, Argentina; c Department of Surgery, George Washington University, Washington, DC, USA

**Keywords:** One-anastomosis gastric bypass, Bile reflux, Roux-en-Y gastric bypass, Conversion surgery

## Abstract

**Introduction:**

Bile reflux is a recognized complication of One-Anastomosis Gastric Bypass (OAGB). Its management can be challenging, particularly when symptoms persist despite medical therapy or surgical diversion.

**Case presentation:**

A 46-year-old woman developed persistent bilious vomiting, nocturnal regurgitation, and aspiration following OAGB. Preoperative imaging indicated gastroesophageal reflux and a recurrent hiatal hernia. The patient underwent conversion to laparoscopic Roux-en-Y gastric bypass (LRYGB) due to her persistent symptoms and related nutritional impairment. Postoperative follow-up indicated total symptom relief and nutritional recovery thanks to the creation of the entero-enteric anastomosis.

**Discussion:**

This case illustrates the outstanding efficacy of entero-enteric diversion in specific individuals and emphasizes the importance of LRYGB in addressing refractory bile reflux, especially in the presence of anatomical considerations like hiatal hernia. The anatomical alteration of LRYGB provides functional segregation of biliopancreatic secretions, inhibiting their reflux into the esophagus.

**Conclusion:**

Converting to LRYGB effectively addresses chronic biliary reflux following OAGB. In analogous situations, the evaluation of revisional surgery may save further esophageal injury and enhance quality of life.

## Highlights

• Persistent bile reflux after OAGB is hard to treat with medical or intermediate surgical options.
• This case illustrates the success of entero-enteric diversion to resolve bile reflux symptoms post-OAGB.
• Recurrent hiatal hernia may worsen bile reflux after OAGB and should be assessed.
• LRYGB should be considered for refractory bile reflux to prevent esophageal damage.

## 1 Introduction

Bariatric surgery has become a cornerstone in the treatment of severe obesity and its associated metabolic disorders. The One-Anastomosis Gastric Bypass (OAGB), was described in 1997 by Rutledge et al. [1]. Among the available surgical alternatives, it has emerged as an increasingly popular procedure due to its technical simplicity, shorter operative time, and promising outcomes regarding weight loss and resolution of comorbidities. However, despite its growing use, OAGB is associated with a distinct set of complications. Bile reflux is one of the most significant concerns as a complication, given its potential to impair quality of life and cause damage to the upper gastrointestinal tract, particularly the esophagus [2]. While various strategies have been proposed to manage bile reflux, including medical therapy and surgical revisions, these approaches are not always successful.

In this report, we present the case of a patient who developed persistent and symptomatic bile reflux following OAGB. Finally, a conversion to laparoscopic Roux-en-Y gastric bypass (LRYGB), characterized by the creation of an entero-enteric anastomosis that diverted bile away from the gastric pouch, resulted in complete resolution of symptoms and nutritional recovery. This case highlights the complexity of bile reflux in patients post-OAGB and reinforces the role of LRYGB as a definitive solution in selected patients. This work has been reported in line with the SCARE criteria [3].

## 2 Case presentation

A 46-year-old woman presented with a one-year history of progressive gastroesophageal symptoms, including nocturnal regurgitation, aspiration, and postprandial bilious vomiting. She had gastric sleeve surgery in 2014, which was later revised due to suboptimal symptom control and weight trajectory. In 2022, she underwent surgical repair of a hiatal hernia. The following year, in 2023, she was converted to an OAGB. Despite this conversion, the patient developed significant bile reflux. Her medical history was notable for hypertension and hypothyroidism, managed with valsartan, amlodipine, and levothyroxine.

**Fig. 1:**
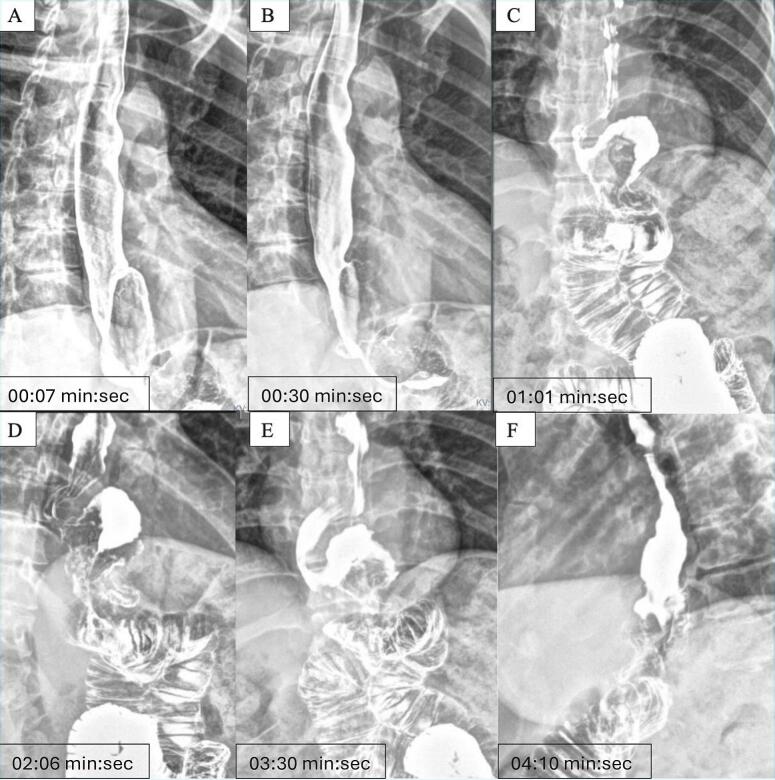
A-F. Upper gastrointestinal series (UGI) – double-contrast technique. A small gastric pouch is visible with appropriate transit of contrast medium into the jejunal limb. Retrograde contrast reflux is observed reaching the mid-esophagus, indicating significant gastroesophageal reflux before the entero-enteric diversion approach. Time labels have been included in each image from the moment of contrast ingestion.

**Fig. 2:**
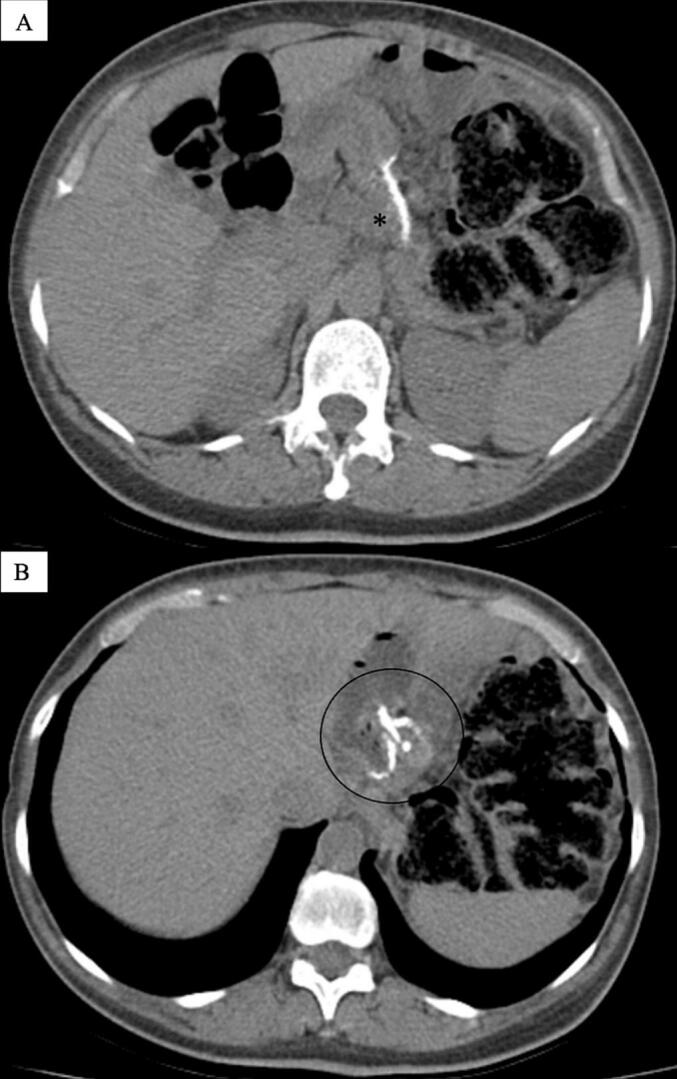
Preoperative imaging of OAGB configuration. (A) Axial view demonstrating the mechanical gastrojejunal anastomosis in the characteristic omega-loop configuration of OAGB (*). (B) Metallic staples delineate the gastric pouch in proximity to the biliopancreatic limb. These images were obtained prior to conversion to LRYGB.

Persistent symptoms led to further evaluation. An upper gastrointestinal contrast series using double-contrast technique showed a small gastric pouch with unobstructed flow of contrast into the jejunal limb (Fig. 1). However, active gastroesophageal reflux was observed, with retrograde contrast reaching the mid-esophagus. An upper endoscopy revealed inflammatory changes consistent with esophagitis and evidence of a recurrent hiatal hernia. Abdominal CT scan (CT) report was reviewed, ruling out obstructive pathology or other significant anatomical abnormalities in the upper gastrointestinal tract (Fig. 2).

## 3 Results

The patient was continuously followed by a bariatric dietitian and underwent behavioral dietary counseling. Despite undergoing surgery and multiple medical therapy procedures, the patient continued reporting bilious vomiting typically occurring two hours after meals. The vomitus was consistently described as bitter, yellow-green in color, and associated with upper abdominal discomfort and early satiety. The symptoms significantly affected her quality of life, and she also expressed episodes of nocturnal regurgitation and coughing suggestive of aspiration.

The imaging studies corroborated her clinical presentation. The upper gastrointestinal series (UGIS) confirmed both the appropriate anatomical configuration of the bypass and the presence of gastroesophageal reflux (Fig. 1), which was deemed pathological due to its severity and height of retrograde contrast reflux. The upper endoscopy confirmed inflammation of the esophageal mucosa and a recurrent hiatal hernia, both of which may have contributed to her persistent symptoms. Despite the absence of mechanical obstruction, these findings suggested the functional failure of the OAGB to control the gastroduodenal reflux.

**Fig. 3:**
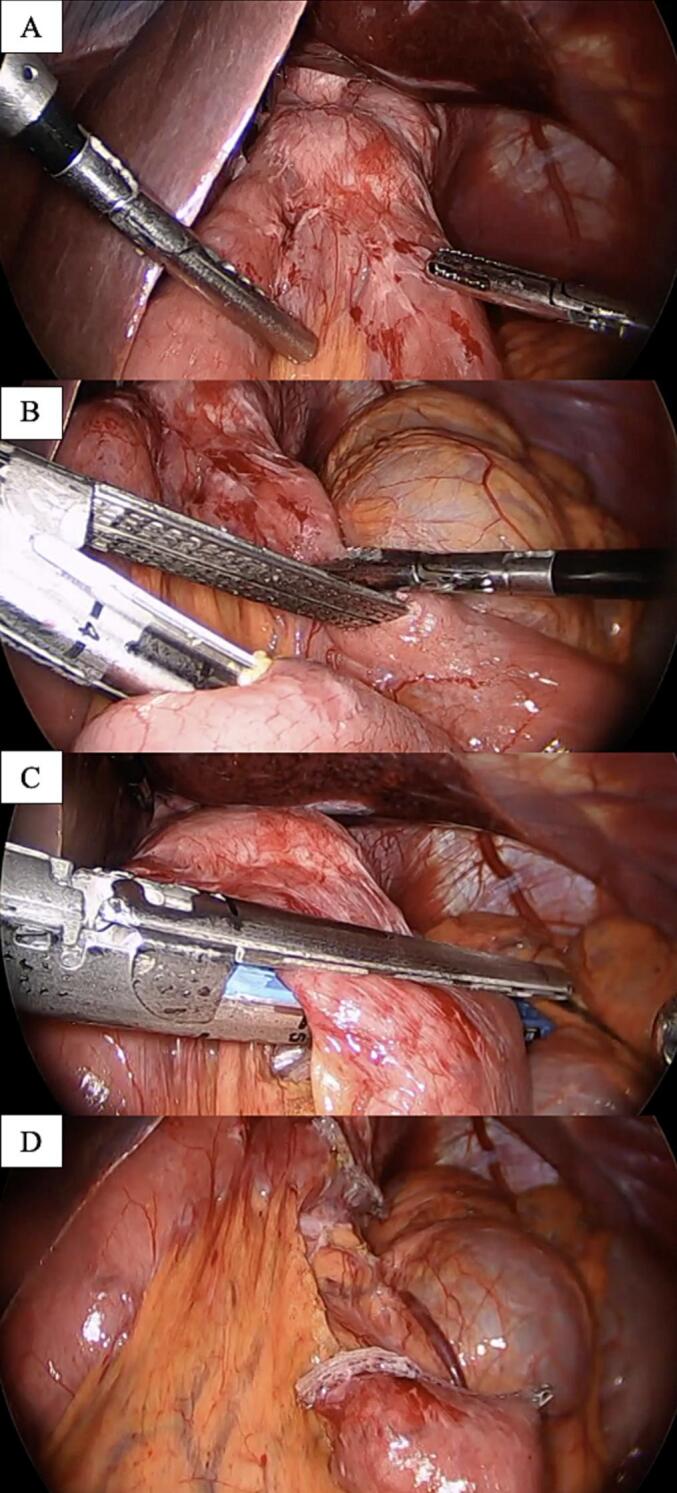
Intraoperative views during conversion to Laparoscopic Roux-en-Y gastric bypass. (A) Identification of the OAGB anatomy with the omega-shaped loop. (B) Creation of a new entero-enteric anastomosis. (C) Isolation of the biliopancreatic limb. (D) Completion of the Roux-en-Y reconstruction with clear separation of biliary and alimentary flow.

Given the refractory nature of her symptoms, in early 2024, the patient underwent conversion from OAGB to LRYGB (Fig. 3). As a result of this surgery, the results of an entero-enteric anastomosis diverted bile away from the esophagus and alleviated her symptoms. Her preoperative weight was 55 kg, with a BMI of 21. Although within normal range, this weight reflected a state of malnutrition driven by persistent vomiting and poor oral intake, especially of protein-rich foods.

The surgery was performed without complications. We used a 36 Fr bougie to check for any leakage. A 45 mm linear stapler was used for the gastrojejunostomy anastomosis and the posterior layer was reinforced with interrupted hand-sewn sutures to ensure adequate sealing and tension control. Anastomosis size on the last gastroscopy was approximately 25 mm. The alimentary limb measured 120 cm and the biliopancreatic limb length was 150 cm.

The postoperative course was outstandingly favorable. At 20 days, she tolerated the progression of a gastric diet without difficulty. By one month postoperatively, she was completely asymptomatic, with no episodes of vomiting or reflux, and successfully transitioned to a structured low-calorie diet. At two months, her weight had increased to 57.7 kg, indicating improved nutritional intake. She reported a broader and more varied diet, with significantly improved tolerance to previously intolerable foods, which had previously triggered immediate emesis. These clinical improvements reflected successful resolution of bile reflux and recovery of gastrointestinal function, directly attributable to the conversion to LRYGB.

Follow-up and imaging of the patient are scheduled at 6 and 12 months respectively, to prevent any complication and to control patient symptoms. In addition, we integrate nutritional laboratories, dietitian and psychologist consultations to have a multidisciplinary treatment.

## 4 Discussion

Bile reflux is a recognized complication after OAGB, with reported rates ranging from 7.8 % to 55.5 %, depending on follow-up duration and diagnostic criteria [4]. While the exact mechanism is multifactorial, the single-loop reconstruction in OAGB inherently allows duodenal contents, including bile, to reach the gastric pouch and, potentially, the esophagus. This reflux is often resistant to standard acid suppression therapy due to the non-acidic nature of bile. Reflux symptoms have remained a major side effect of OAGB.

Initial management strategies include lifestyle changes, dietary adjustments, and high-dose proton pump inhibitors, although the efficacy of PPI therapy is limited in bile-mediated esophagitis. When medical treatment fails, surgical alternatives such as entero-enteric diversion like conversion to LRYGB are considered [5,6]. Different numbers can be found in the literature when it comes to the need for conversion to LRYGB [6].

It is interesting to point out that the OAGB had superior short-term weight loss and a lower complications profile compared to LRYGB [7]. Still, we must acknowledge when it should be the indicated surgery for the patient and opt for the LRYGB when it is appropriate.

A systematic review and meta-analysis by Sargsyan et al. showed that the most common indication for conversion surgery in patients with OAGB was biliary reflux (47.8 %) like in our case [5]. We perfectly agree with the study made by Jedamzik et al., which explains that all patients under conversion to LRYGB for bile reflux should have previous medical treatment for at least 6 months and additional counseling by a dietologist [6].

Esophageal manometry and gastric scintigraphy may offer valuable preoperative insights, particularly in patients presenting with persistent vomiting, early satiety, or atypical symptoms. Manometry allows for the assessment of esophageal motility prior to revisional procedures that may further impact gastrointestinal transit, while gastric scintigraphy provides objective data on gastric emptying and can help rule out conditions such as gastroparesis. As mentioned by Felsenreich et al., these functional studies are especially useful when planning conversion from OAGB to LRYGB in patients with severe symptomatology [8]. In our case, these investigations were not performed due to limited local availability; however, we acknowledge their potential utility in selected patients.

The physiological basis of LRYGB lies in its separation of the biliopancreatic limb from the alimentary limb, effectively preventing bile from reaching the gastric pouch or esophagus. This anatomical change is particularly valuable in patients with refractory symptoms or anatomical contributors such as hiatal hernia, as in the present case.

In our patient, a recurrent hiatal hernia likely perpetuated bile reflux despite prior revisions. Only after conversion to LRYGB could she achieve a complete symptom resolution with weight recovery and improved food tolerance. This clinical course is strongly aligned with existing literature supporting LRYGB as the most reliable surgical solution for bile reflux after OAGB.

## 5 Conclusion

This case highlights the complexity and clinical relevance of persistent bile reflux following OAGB, even after multiple therapeutic interventions. Ultimately, conversion to Roux-en-Y gastric bypass proved to be the definitive solution, leading to complete symptom resolution and nutritional recovery. In patients with persistent bile reflux unresponsive to conservative and intermediate surgical measures, early consideration of LRYGB may prevent ongoing esophageal injury and improve quality of life. This case supports the role of LRYGB as a highly effective revisional strategy in the management of bile reflux after OAGB, reflecting the function of entero-enteric anastomosis in the final treatment.

## Informed consent

Written informed consent was obtained from the patient for publication of this case report and accompanying images.

## Ethical approval

Ethical approval was not required for this case report in accordance with our institution's policy.

## Guarantor

Andres Fontaine-Nicola, MD.

## Patient perspective

The patient expressed satisfaction with the resolution of symptoms and the quick postoperative recovery, highlighting the relief from her biliary vomits, bringing a better quality of life and improvement of the food processing.

## Author contributions

AFN contributed to the concept, data collection, drafting, and final manuscript editing. AFN, PC, KOS, GC and PO contributed to case management, critical revision, and final approval. All authors read and approved the final manuscript.

## Funding

This research did not receive any specific grant from funding agencies in the public, commercial, or not-for-profit sectors.

## Declaration of Generative AI in writing

During the preparation of this work, the authors did not use artificial intelligence (AI) to assist in language clarity and manuscript organization.

## Declaration of competing interest

None.
